# Microbial magnetite oxidation via MtoAB porin-multiheme cytochrome complex in *Sideroxydans lithotrophicus* ES-1

**DOI:** 10.1128/aem.01865-24

**Published:** 2025-03-05

**Authors:** Jessica L. Keffer, Nanqing Zhou, Danielle D. Rushworth, Yanbao Yu, Clara S. Chan

**Affiliations:** 1 Department of Earth Sciences, University of Delaware 5972 , Newark, Delaware, USA; 2 School of Marine Science and Policy, University of Delaware 5972 , Newark, Delaware, USA; 3 Department of Chemistry and Biochemistry, University of Delaware 5972 , Newark, Delaware, USA; University of Nebraska-Lincoln, Lincoln, Nebraska, USA

**Keywords:** magnetite, iron oxidizers, *Sideroxydans*, Gallionellaceae, multiheme cytochromes, microbe-mineral interactions

## Abstract

Most of Earth’s iron is mineral-bound, but it is unclear how and to what extent iron-oxidizing microbes can use solid minerals as electron donors. A prime candidate for studying mineral-oxidizing growth and pathways is *Sideroxydans lithotrophicus* ES-1, a robust, facultative iron oxidizer with multiple possible iron oxidation mechanisms. These include Cyc2 and Mto pathways plus other multiheme cytochromes and cupredoxins, and so we posit that the mechanisms may correspond to different Fe(II) sources. Here, *S. lithotrophicus* ES-1 was grown on dissolved Fe(II)-citrate and magnetite. *S. lithotrophicus* ES-1 oxidized all dissolved Fe^2+^ released from magnetite and continued to build biomass when only solid Fe(II) remained, suggesting it can utilize magnetite as a solid electron donor. Quantitative proteomic analyses of *S. lithotrophicus* ES-1 grown on these substrates revealed global proteome remodeling in response to electron donor and growth state and uncovered potential proteins and metabolic pathways involved in the oxidation of solid magnetite. While the Cyc2 iron oxidases were highly expressed on both dissolved and solid substrates, MtoA was only detected during growth on solid magnetite, suggesting this protein helps catalyze oxidation of solid minerals in *S. lithotrophicus* ES-1. A set of cupredoxin domain-containing proteins were also specifically expressed during solid iron oxidation. This work demonstrated that the iron oxidizer *S. lithotrophicus* ES-1 utilized additional extracellular electron transfer pathways when growing on solid mineral electron donors compared to dissolved Fe(II).

**IMPORTANCE:**

Mineral-bound iron could be a vast source of energy to iron-oxidizing bacteria, but there is limited physiological evidence of this metabolism, and it has been unknown whether the mechanisms of solid and dissolved Fe(II) oxidation are distinct. In iron-reducing bacteria, multiheme cytochromes can facilitate iron mineral reduction, and here, we link a multiheme cytochrome-based pathway to mineral oxidation, expanding the known functionality of multiheme cytochromes. Given the growing recognition of microbial oxidation of minerals and cathodes, increasing our understanding of these mechanisms will allow us to recognize and trace the activities of mineral-oxidizing microbes. This work shows how solid iron minerals can promote microbial growth, which, if widespread, could be a major agent of geologic weathering and mineral-fueled nutrient cycling in sediments, aquifers, and rock-hosted environments.

## INTRODUCTION

To microbes, minerals provide surfaces to live on, a source of nutrients, and in some cases, a substrate for respiration, for example, for Fe(III)- and S(0)-reducing organisms. We are increasingly finding that microbes can also oxidize minerals, particularly iron minerals such as magnetite (1–3), green rust (4), pyrite (5), biotite (6), and smectites (6–8), using these as a source of electrons and therefore energy. To use minerals as electron donors, cells must be able to transfer electrons from outside the cell to the interior. This capability, known as extracellular electron uptake (EEU), has been demonstrated not only in cultures with minerals but also by experiments on cathodes in bioelectrochemical systems, which provide a continuous supply of electrons directly to colonizing cells (9–13). EEU is a capability of iron-oxidizing bacteria (FeOB), which need to keep iron outside of cells to prevent various detrimental reactions from occurring in the periplasm or cytoplasm (14, 15). Most work on FeOB has focused on oxidation of dissolved Fe^2+^, but if this EEU capability can be adapted to oxidize solid minerals, it would give an energetic advantage, given that most of Earth’s iron is mineral-bound.

However, we do not know how common mineral oxidation is among microorganisms. To recognize and track mineral oxidation, we need to unravel the mechanisms, that is, the genes and proteins involved. This requires an organism that can grow both on dissolved and solid substrates. Among the few reliable chemolithotrophic FeOB isolates, the Gallionellaceae *Sideroxydans lithotrophicus* ES-1 stands out as having a versatile metabolism, able to grow by oxidizing dissolved Fe^2+^, Fe(II)-smectite clays, and thiosulfate (7, 16–18). *Sideroxydans* species have been identified in many environments, including a variety of sediments (19, 20); brackish, freshwater, or groundwater systems (16, 21–30); and rice paddies or other wetlands (31–33), suggesting this genus is highly adaptable, likely linked to its metabolic versatility.

*S. lithotrophicus* ES-1 has a closed, sequenced genome that encodes multiple possible enzymatic pathways for iron oxidation (17, 34, 35). The genome encodes three isoforms of the iron oxidase Cyc2, a fused monoheme cytochrome-porin which has been biochemically demonstrated to oxidize dissolved Fe^2+^ (36, 37). It also encodes MtoAB which is homologous to the iron-oxidizing PioAB complex of the photoferrotroph *Rhodopseudomonas palustris* TIE-1 (38) and the iron-reducing complex MtrAB in *Shewanella* species (39, 40). Porin-cytochrome complexes like MtrAB and PioAB form conduits across the outer membrane, so are key in iron-reducer interactions with minerals (MtrAB [41, 42]) and in oxidation of a cathode (PioAB [43]). Given the predicted structural similarity to these other systems and that heterologously expressed MtoA has been shown to oxidize Fe(II) (44), MtoAB could also play a role in oxidation of extracellular minerals. The genome of *S. lithotrophicus* ES-1 also encodes other porin-cytochrome complexes with large multiheme cytochrome (MHC) subunits and a plethora of heme motif (CXXCH)-containing proteins including probable periplasmic electron carriers (34, 45). Thus, *S. lithotrophicus* ES-1 appears well-endowed with multiple potential iron oxidation and other EEU mechanisms, though it is not certain which ones enable oxidation of minerals.

Recent work on *S. lithotrophicus* ES-1 demonstrated for the first time the ability of this organism to utilize a solid Fe(II) source for growth and gave us some initial clues to the possible mineral oxidation mechanism (7). The porin MtoB was detected in cells grown on Fe(II)-smectite clays but not dissolved Fe(II)-citrate. The MHC MtoA was not observed, possibly because MHCs can be difficult to detect by mass spectrometry due to the large number of covalently modified cysteines per peptide length. The proteomics was supplemented with reverse transcription quantitative PCR (RT-qPCR), which confirmed that *mtoA* was upregulated on smectite compared to Fe(II)-citrate. This led to the hypothesis that in *S. lithotrophicus* ES-1, the MtoAB complex plays a specific role in oxidation of solid iron minerals, but not aqueous Fe(II)-citrate (7). However, given that only a limited proportion of proteins (<25% of total proteome) were detected in this study, improvements to enhance proteome coverage for low-input samples are necessary to accurately distinguish proteins expressed on solid substrates.

Incomplete proteomes can result from low biomass input, as can often be the case for FeOB, since cultures are challenging. In the smectite study of *S. lithotrophicus* ES-1, large volumes of cultures were required to obtain enough cells for molecular analyses such as proteomics (7). Recently, this need for large culture volumes was eliminated with the development of a novel on-filter in-cell (OFIC) processing pipeline for proteomic analyses of low biomass samples (46–48). This single-vessel method avoids cell lysis, which tends to cause significant sample loss particularly for low-input samples and performs all the treatments in the same filter device, thus drastically simplifies sample preparation and improves proteomic sensitivity. In a pilot study, ~76% of the entire *S. lithotrophicus* ES-1 proteome was identified from just 10 mL of culture (~1 × 10^9^ cells) (46).

Minerals with high Fe(II) content commonly interfere with molecular extractions, making it difficult to obtain complete “omics” data sets. In the smectite study, clays interfered with downstream analyses (7), so we investigated the possibility of using magnetite, which can be easily removed from cultures with a magnet. As a mixed-valence iron mineral (Fe^II^Fe^III^_2_O_4_) common in sediments (49), magnetite could potentially serve as an electron donor to support the growth of Fe(II)-oxidizing bacteria. We hypothesized *S. lithotrophicus* ES-1 could grow by oxidizing Fe(II) in magnetite, in part because *S. lithotrophicus* ES-1 grows on other iron minerals and also based on previous observations of other FeOB that were able to oxidize magnetite. The photoferrotroph *R. palustris* TIE-1 oxidized chemically synthesized magnetite (1, 50), while nitrate-reducing Fe(II)-oxidizers including *Acidovorax* sp. 2AN and the enrichment culture KS have been observed to oxidize biogenic magnetite (2, 3). If *S. lithotrophicus* ES-1 is able to oxidize magnetite, this would give us an optimal system for investigating proteins involved in solid Fe(II) oxidation.

Here, we tested *S. lithotrophicus* ES-1 growth on three batches of abiogenic magnetite (two synthesized in house and one purchased from a commercial vendor) and compared protein expression to cells grown on dissolved Fe^2+^. The substrates differed in particle size, crystallinity, and solubility, which allowed us to evaluate growth and Fe(II) oxidation mechanisms in the presence of different proportions of solid and dissolved Fe^2+^. This work gives further evidence that FeOB can grow by oxidizing mineral-bound Fe(II) along with insight into the mechanisms that enable electron uptake from solids.

## RESULTS

### Magnetite characterization

We characterized the magnetites to determine particle size, crystallinity, and solubility. The X-ray diffraction (XRD) patterns of fresh synthetic magnetite, aged synthetic magnetite, and commercial magnetite all possessed peaks characteristic of magnetite (Fig. S1). The sharp, narrow peaks in the commercial magnetite XRD pattern indicate the particles are more crystalline, and the particle size is calculated to be ~27 nm. The synthetic magnetites have broader peaks in their XRD patterns, indicating lower crystallinity/smaller domain size, with the fresh synthetic magnetite having the smallest size (<10 nm).

**Fig 1:**
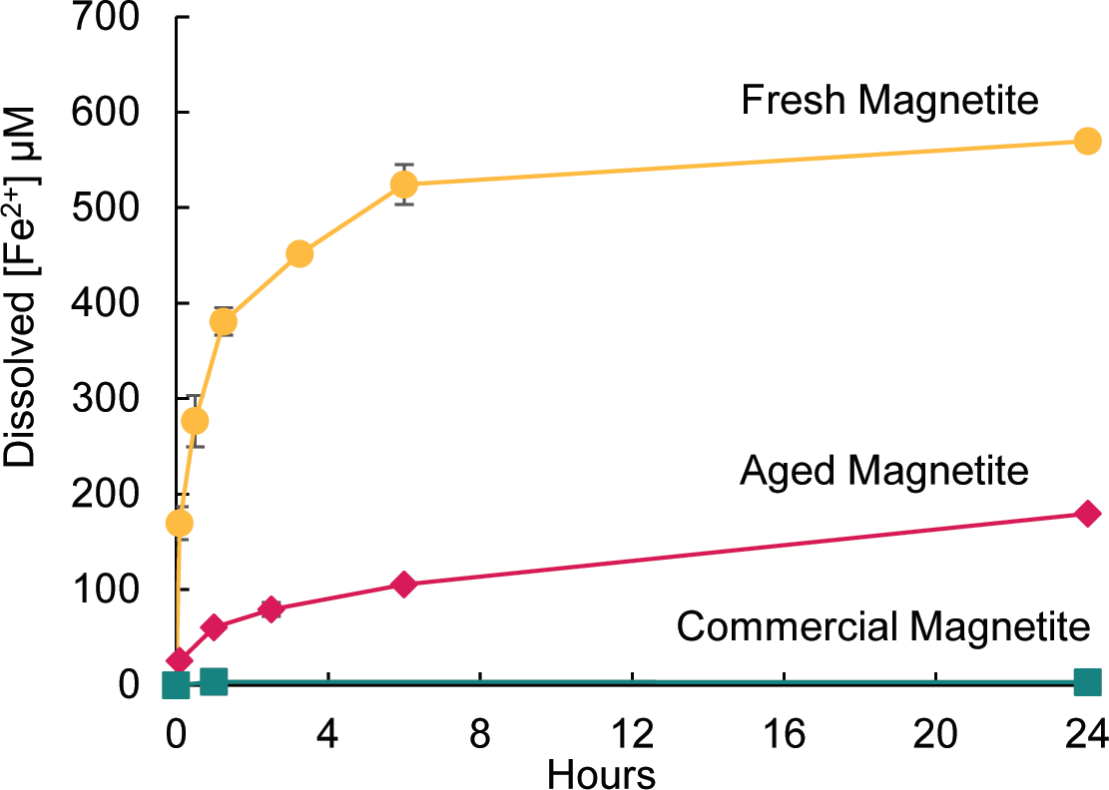
Dissolved Fe^2+^ released under anoxic conditions in 20mM MES pH 6.0 from different magnetite types: fresh synthetic magnetite (gold; circles), aged synthetic magnetite (pink; diamonds), commercial magnetite (teal; squares). Error bars are ±1 standard deviation of replicates.

Nanocrystalline minerals tend to be more soluble (51, 52), and this was confirmed by suspending 1 g/L (12.9 mM Fe) of magnetite particles in anoxic 20 mM 2-(N-morpholino)ethanesulfonic acid (MES) buffer (pH 6.0) and measuring dissolved Fe^2+^ over the course of 24 hours (Fig. 1). The fresh synthetic magnetite was the most soluble, releasing a maximum of 570 µM Fe^2+^ [~13% of total Fe(II)], which fits with the lower crystallinity of this phase. The aged synthetic magnetite was less soluble, releasing at most 179 µM Fe^2+^ [~4% of total Fe(II)], and the commercial magnetite was the least soluble at <10µM Fe^2+^ [the limit of detection in the assay; <0.2% of total Fe(II)]. To estimate the dissolved Fe^2+^ released from the synthetic magnetites over a longer time in the absence of cells, dissolved Fe^2+^ concentrations were measured at 24-hour intervals in incubations using either anoxic buffer or buffer equilibrated with 2% oxygen to simulate the conditions for culturing. The buffer was then replaced with fresh solution to remove all dissolved Fe^2+^, and dissolved Fe^2+^ was remeasured after an additional 24 hours, and the process was repeated once more. Each day, the dissolved Fe^2+^ release decreases, implying there is less soluble Fe(II) available over time. By the third incubation, the dissolved Fe^2+^ released from the fresh and aged synthetic magnetites was <100 µM (Fig. S2). Having magnetites of different solubilities allows us to evaluate growth and mineral oxidation mechanisms in the presence of different amounts of dissolved Fe^2+^, covering a range of possible environmental scenarios.

### *S. lithotrophicus* ES-1 growth on magnetite

**Fig 2:**
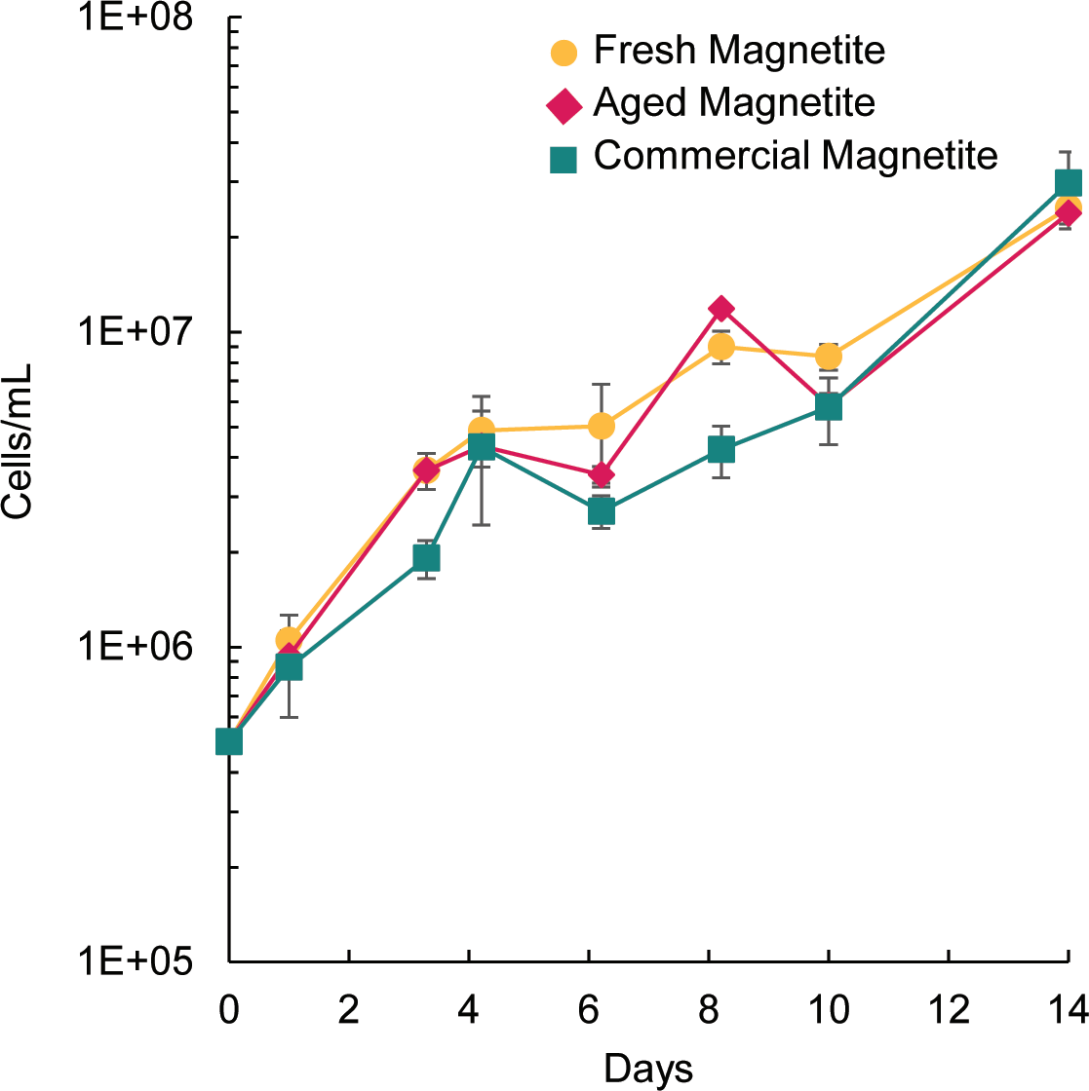
*S. lithotrophicus* ES-1 growth on fresh synthetic magnetite (gold; circles), aged synthetic magnetite (pink; diamonds), and commercial magnetite (teal; squares). Error bars are ±1 standard deviation of replicates.

Culturing experiments demonstrated that all magnetites supported growth of *S. lithotrophicus* ES-1. Over the course of a 14-day incubation, the cell numbers increased ~50-fold in bottles containing all types of magnetite (Fig. 2; fresh magnetite 49.5×; aged magnetite 47.7×; commercial magnetite 59.4×). Cell numbers increased faster on the fresh and aged synthetic magnetites than on the commercial magnetite during the first 4 days, but at the end of the experiment, cell numbers were similar in all conditions (Fig. 2). The final cell yield of ~2–3 × 10^7^ cells/mL is similar to the cell yield observed when *S. lithotrophicus* ES-1 was grown on 1 g/L of Fe(II)-smectite clay (7).

In previous experiments, we observed that *S. lithotrophicus* ES-1 experienced exponential growth for 5 days with a maximum cell yield of <2 × 10^6^ cells/mL when provided with only 100µM dissolved Fe^2+^ per day (in the form of Fe(II)-citrate) (17). In the magnetite cultures, *S. lithotrophicus* ES-1 reached more than one order of magnitude higher cell density and continued to build biomass through day 14 (Fig. 2), long after the available dissolved Fe^2+^ dropped below 100 µM (Fig. S2), suggesting *S. lithotrophicus* ES-1 is either promoting magnetite dissolution or accessing the solid magnetite directly.

### Dissolved and solid iron oxidation

**Fig 3:**
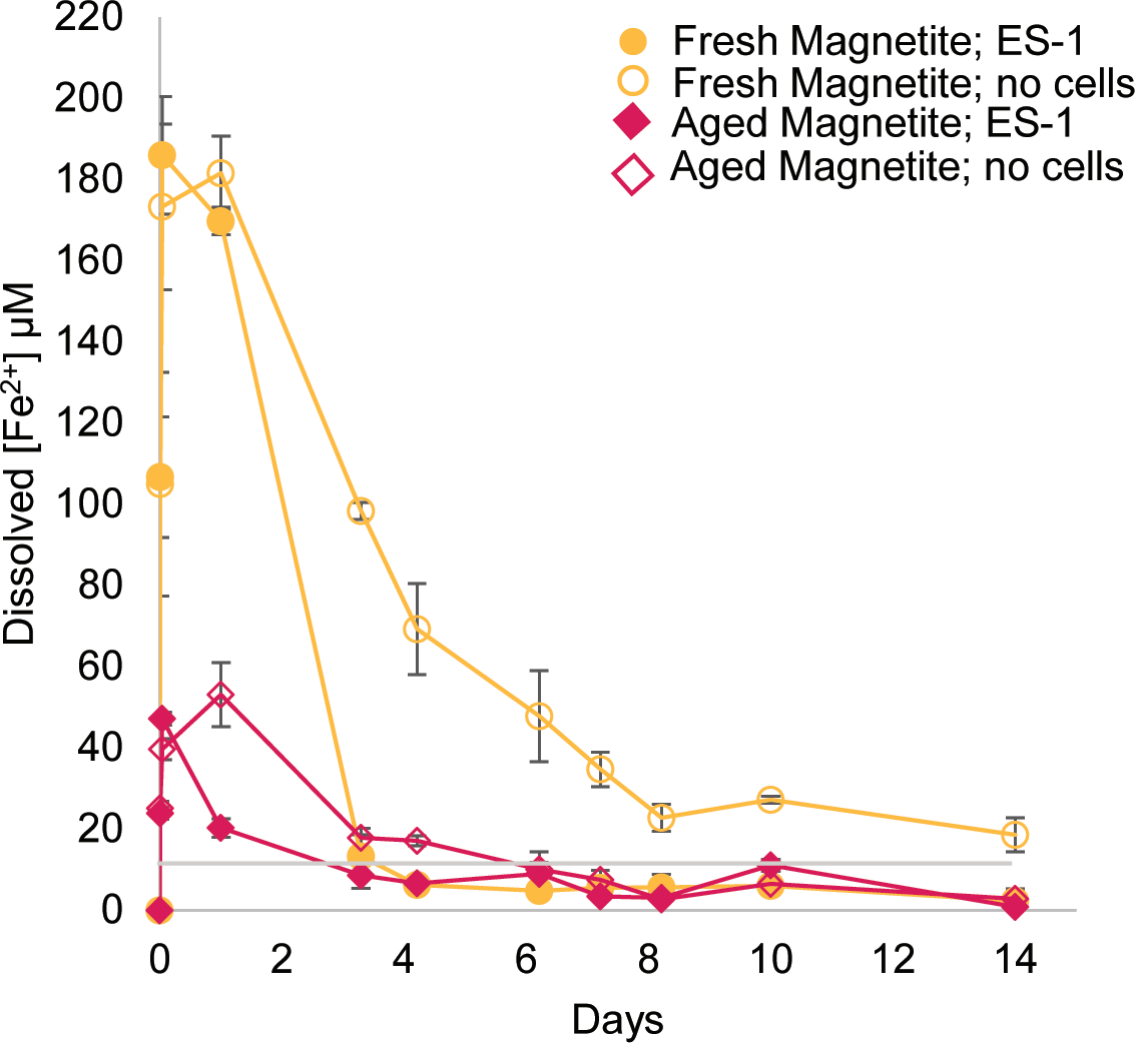
Dissolved Fe^2+^ remaining in cultures with *S. lithotrophicus* ES-1 (filled) or no cell controls (open) with different magnetite types: fresh synthetic magnetite (gold; circles) and aged synthetic magnetite (pink; diamonds). Commercial magnetite measurements were always <10 µM and were not plotted. Gray line at 10 µM is detection limit. Error bars are ±1 standard deviation of replicates.

We tracked the dissolved Fe^2+^ and Fe(II)/Fe(III) in magnetite to evaluate whether *S. lithotrophicus* ES-1 was oxidizing one or both forms of iron. In *S. lithotrophicus* ES-1 cultures with either fresh synthetic magnetite or aged synthetic magnetite, all dissolved Fe^2+^ was oxidized in the culture within 3 days (Fig. 3). The rate of oxidation of dissolved Fe^2+^ by oxygen was slower in the bottles without cells: in the fresh synthetic magnetite bottle, measurable dissolved Fe^2+^ remained at the end of the experiment, while in the aged synthetic magnetite bottle, dissolved Fe^2+^ was measurable until day 6. Because biotic oxidation is faster than the abiotic controls, *S. lithotrophicus* ES-1 cells are accelerating and/or catalyzing iron oxidation. In commercial magnetite bottles both with and without *S. lithotrophicus* ES-1, concentrations of dissolved Fe^2+^ were <10 μM at all time-points, suggesting that growth could be based primarily on oxidation of solid magnetite.

**Fig 4:**
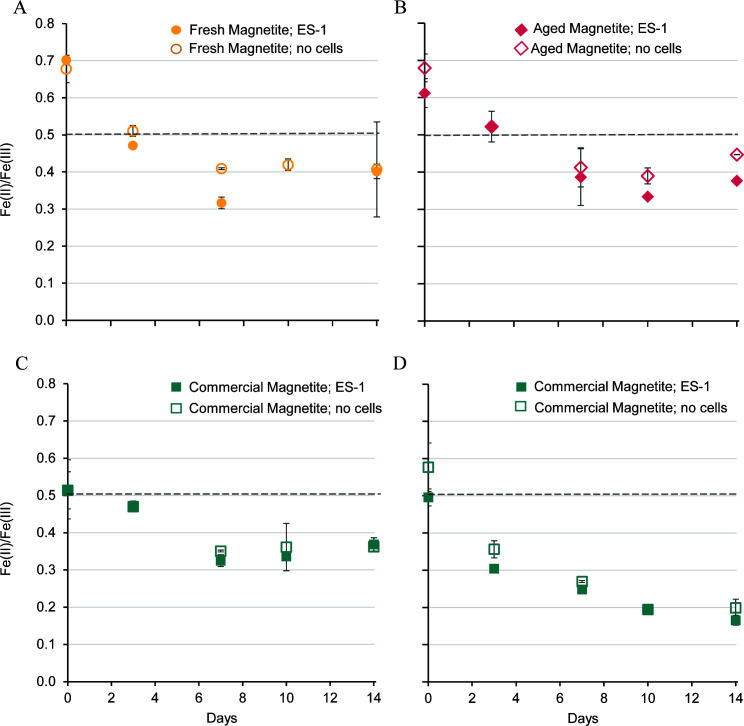
Measurements of the Fe(II) to Fe(III) ratio in acid-dissolved solid magnetite particles from cultures with *S. lithotrophicus* ES-1 (filled) or no cell controls (open) with different magnetite types: (A) fresh synthetic magnetite; (**B**) aged synthetic magnetite, (**C**) commercial magnetite, full dissolution (6 M HCl; 24 hours), and (D) commercial magnetite, partial dissolution (1 M HCl; 1 hour). Dashed line indicates the stoichiometric magnetite ratio. Error bars are ±1 standard deviation of replicates.

The Fe(II)/Fe(III) content of the magnetite was measured in minerals sampled over the course of the experiment (Fig. 4). At the start of the experiment, both of the synthetic magnetites were more reduced (Fe(II)/Fe(III) = 0.6–0.7) than stoichiometric magnetite (Fe(II)/Fe(III) = 0.5). In cultures with *S. lithotrophicus* ES-1, fresh synthetic magnetite was more oxidized (a lower Fe(II)/Fe(III) ratio) on day 7 compared to the abiotic bottles (*P* < 0.005); however, by the end of the experiment, ratios measured for both bottles were similar (Fig. 4A). In the aged synthetic magnetite bottles, there was more oxidation in the bottles with *S. lithotrophicus* ES-1 compared to bottles without cells starting at day 10 (*P* < 0.005 at day 14; Fig. 4B). In contrast, for the commercial magnetite bottles, there was no difference in the ratio between bottles with cells and without cells. Despite the little measurable difference between rates of abiotic and biotic oxidation of solid magnetite, *S. lithotrophicus* ES-1 is capable of growing in the presence of solid magnetite as the sole electron donor. This suggests even if the cells do not accelerate the rate of oxidation as measured at the bulk level, they are still able to outcompete O_2_ for electrons. By day 14, the different types of magnetite were oxidized to a similar extent (Fe(II)/Fe(III) ~0.4; Fig. 4), despite their various initial sizes, crystallinities, and starting Fe(II)/Fe(III) ratios. This suggests there is a proportion of Fe(II) in each of the magnetite structures that is inaccessible to either microbes or oxygen under these culture conditions.

Because these were bulk measurements, it was possible there was preferential oxidation of the surface that was obscured. To address this, the solid commercial magnetite particles were subjected to a partial dissolution step (~15% dissolved in 1 M HCl) to measure reactive Fe(II) and Fe(III) of the surface. These results indicated that there was more oxidation of the surface (Fig. 4D) compared to the bulk particles (Fig. 4C), although there was still not much difference between cultures with *S. lithotrophicus* ES-1 and no-cell control bottles. Combining the results from all measurements of dissolved Fe^2+^ and Fe(II)/Fe(III) in magnetite suggests the dissolved Fe^2+^ is quickly oxidized by the microbes (Fig. 3), and the microbes could be concurrently accessing electrons from solid Fe(II) since the magnetite Fe(II)/Fe(III) ratio decreases by the first measurement on day 3 (Fig. 4).

### Proteome analyses

Quantitative proteomic analyses were performed to explore *S. lithotrophicus* ES-1 iron oxidation mechanisms on aqueous Fe(II) and magnetite. Magnetite cultures (Fig. 2) were compared to Fe(II)-citrate cultures (Fig. S3) at an early growth time-point (day 3 for the magnetites or day 2 for Fe(II)-citrate) or a late growth time-point (day 14 for magnetites or day 7 for Fe(II)-citrate). *S. lithotrophicus* grew faster on Fe(II)-citrate, but the cell number was normalized during collection for the proteome analysis. A total of 2,309 out of 2,978 proteins encoded in the genome (~78%) were identified across all eight conditions (832–2068 proteins per sample), from 10 to 100 mL of culture, demonstrating that the OFIC processing method used here is a significant improvement over the previous proteomic pipelines (7) for low biomass samples.

**Fig 5:**
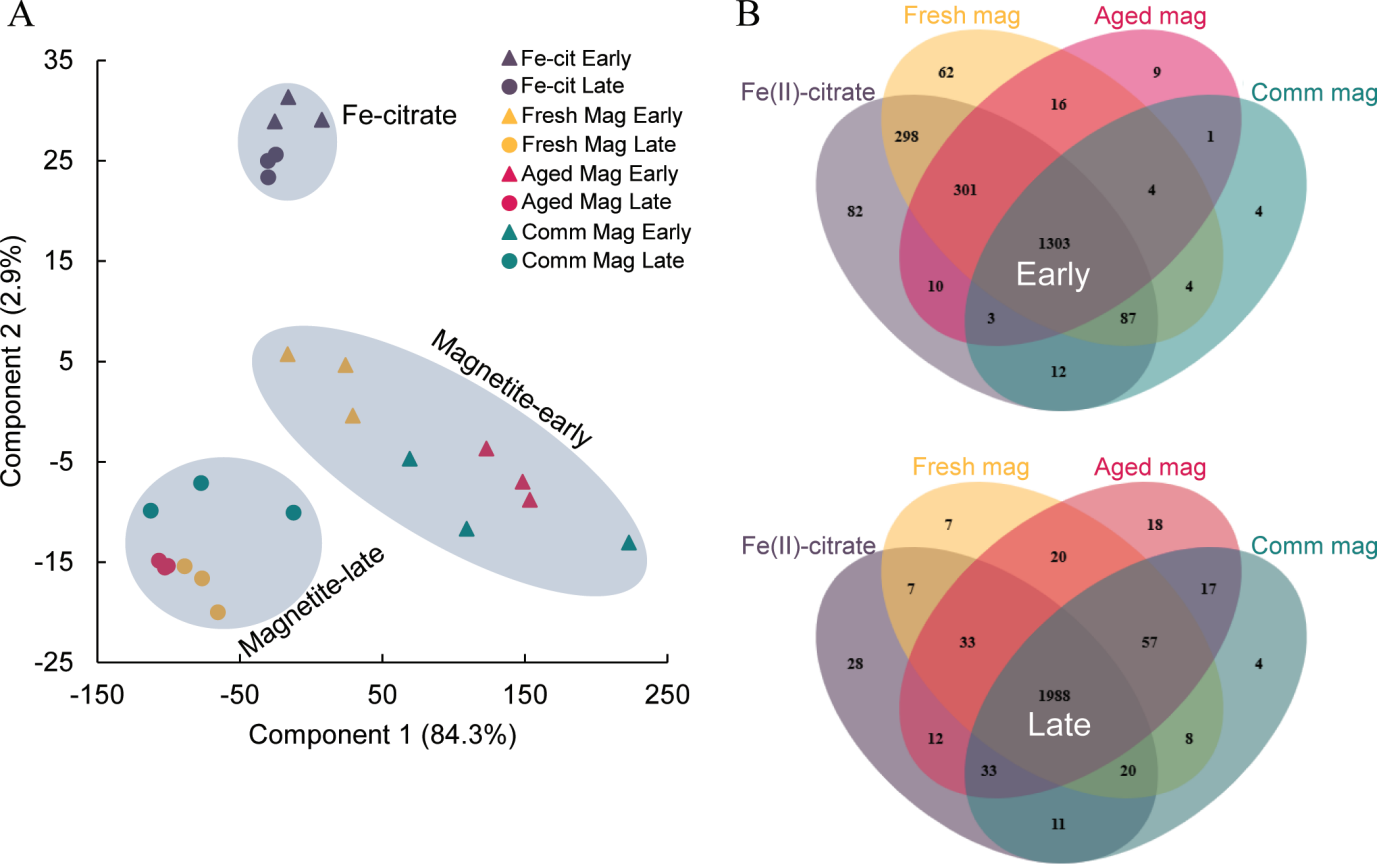
(**A**) PCA plot of the different sample types. Early time-points (triangles), late time-points (circles), Fe(II)-citrate (purple), fresh synthetic magnetite (gold), aged synthetic magnetite (pink), and commercial magnetite (teal). (**B**) Venn diagrams showing the number of proteins identified in each condition for the early time-point (top) and the late time-point (bottom). Fe-cit, Fe(II)-citrate; Mag, magnetite; Comm, commercial.

Principal component analyses (PCA) showed that all of the Fe(II)-citrate-grown samples were most similar to one another (Fig. 5A); these two time-points shared 93% of the proteins detected. There was a clear separation of the Fe(II)-citrate and magnetite samples along the component 2 axis (Fig. 5A), while the early and late time-point samples of the magnetites were separated along the component 1 axis. The magnetite samples showed more differentiation in the PCA, though all six magnetite samples did share 91% of the proteins detected, suggesting the magnetite-grown cultures express a core set of proteins. Together, these results show that the growth phase and type of available Fe(II) source exert large influences on the variation within the protein expression profiles.

The fresh and aged synthetic magnetite cultures were more similar to the Fe(II)-citrate cultures at the early time-point. There were 298 proteins shared between the Fe(II)-citrate and fresh synthetic magnetite cultures that were not present in any other culture (Fig. 5B), and a pair-wise comparison found no statistically significant differences in protein abundances of the shared proteins between these two conditions. An additional 301 proteins were shared between the early time-point fresh synthetic, aged synthetic, and Fe(II)-citrate cultures. These cultures were the only ones with measurable amounts of dissolved Fe^2+^ (Fig. 3); thus, the shared proteins may represent mechanisms and adaptations for utilizing dissolved Fe^2+^.

**Fig 6:**
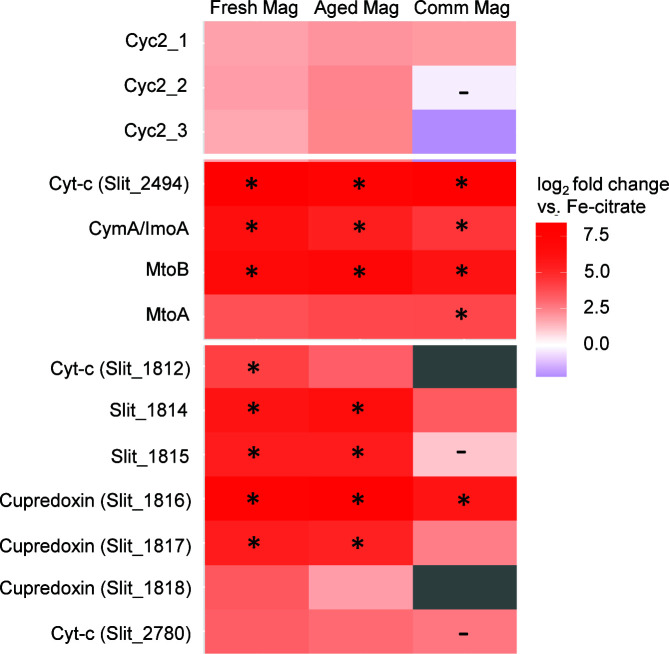
Heatmap of the log_2_ fold change in expression between each of the late magnetite samples and the late Fe(II)-citrate sample. Boxes marked with an asterisk (*) are within the top 1% of most differentially expressed proteins. All comparisons are statistically significant (P_adj_ < 0.05), with the exception of boxes marked with a dash (-). Gray boxes indicate protein was not detected in at least one comparison condition. Mag, magnetite; Comm, commercial.

*S. lithotrophicus* ES-1 encodes two distinct Fe(II) oxidation pathways, the MtoAB complex and Cyc2 (17, 34–36, 44). The Mto complex is comprised of a periplasmic decaheme MtoA (Slit_2497) and an outer membrane porin MtoB (Slit_2496) (35, 44). The same gene cluster encodes an inner membrane tetraheme protein CymA/ImoA (Slit_2495) and a periplasmic monoheme protein (MtoD; Slit_2498) (35, 53, 54). MtoA and CymA/ImoA were only detected in the magnetite cultures but not in the Fe(II)-citrate culture (Table 1) and were among the most differentially expressed proteins (Fig. 6; Table S1). The Mto complex was expressed even in the early magnetite cultures, suggesting the expression of Mto is controlled by the presence of the solid magnetite, regardless of the presence of dissolved Fe^2+^. These results are in agreement with the previous study with *S. lithotrophicus* ES-1 growing either on dissolved Fe(II)-citrate or on solid Fe(II)-smectite clays, in which some of the proteins of the Mto complex were only detected in the solid Fe(II) cultures (7).

**TABLE 1:** Maximum percentile of protein expression based on iBAQ values^*d*^

Protein name	Locus tag	Fe-cit^*a*^ (early)	Fe-cit^*a*^ (late)	Fresh Mag^*b*^ (early)	Fresh Mag^*b*^ (late)	Aged Mag^*b*^ (early)	Aged Mag^*b*^ (late)	Comm Mag^*b*^^*,^c^*^ (early)	Comm Mag^*b*^^*,^c^*^ (late)
Cyc2_1	Slit_0263	99.8	99.7	99.8	99.8	99.9	99.9	99.7	99.9
Cyc2_2	Slit_0264	96.4	93.6	98.9	95.4	99.1	97.2	95.2	86.3
Cyc2_3	Slit_0265	90.7	85.2	97.8	91.0	95.7	92.8	86.0	45.5
MtoA	Slit_2497	0	0	54.5	44.1	39.9	52.5	48.0	45.0
MtoB	Slit_2496	18.2	8.4	89.9	89.5	83.2	86.5	66.0	84.4
CymA/ImoA	Slit_2495	0	0	62.7	79.5	53.9	76.3	68.6	53.3
Cyt-c (Mto)	Slit_2494	18.0	0	89.7	94.8	88.7	92.8	89.3	94.6
PCC3(porin)	Slit_0867	11.7	15.2	10.5	16.5	0	7.2	0	14.7
PCC3(IMP)	Slit_1446	43.7	54.3	25.6	55.7	60.5	50.9	58.0	60.7
PCC3(cyt-c_p)	Slit_1447	22.2	15.8	20.3	14.9	0	22.4	48.3	15.7
PCC3(cyt-c_e)	Slit_1448	0	0	0	0	0	0	0	0
PCC3(porin)	Slit_1449	48.5	50.6	54.0	60.7	54.0	54.8	50.9	63.4
Cyt b	Slit_1321	69.2	65.3	55.9	50.4	49.9	45.3	0	44.8
Hypothetical	Slit_1322	81.8	76.6	85.3	68.5	87.3	69.9	0	56.8
Cyt-c	Slit_1323	94.4	92.1	92.2	91.4	93.5	92.7	91.7	87.6
Cyt-c	Slit_1324	95.3	93.9	93.4	93.4	93.3	94.2	93.5	91.0
Cyt-c	Slit_1353	96.3	95.6	95.3	97.7	96.9	97.5	95.8	98.2
Cyt-c	Slit_2042	99.3	99.4	99.0	99.5	97.2	99.1	98.7	99.5
Cyc1(cyt-c)	Slit_2657	97.4	96.2	98.7	99.0	99.4	98.9	98.4	99.1
Cyt-c	Slit_2780	0	32.8	42.6	73.9	37.4	47.8	0	65.2
Cupredoxin	Slit_1816	15.3	13.7	21.1	79.3	30.2	73.0	0	57.2
Cupredoxin	Slit_1817	0	0	33.0	74.8	0	62.4	0	42.7
Cupredoxin	Slit_1818	0	0	0	29.5	0	21.2	0	9.0

^*a*^ Fe-cit, Fe(II)-citrate.

^*b*^ Mag, magnetite.

^*c*^ Comm, commercial.

^*d*^ cyt – cytochrome; p – periplasmic; e - extracellular.

Expression of MtoD was not detected in any culture, but a protein encoded by the gene downstream of *cymA/imoA* (UniProt entry: D5CMP7, locus tag: Slit_2494) showed similar expression patterns as the other Mto-related proteins (Fig. 6; Table S1). This protein is poorly annotated but contains one heme binding motif and a transmembrane signal peptide, suggesting it could also be a periplasmic cytochrome involved in the Mto-based iron oxidation pathway.

The other Fe(II) oxidase in *S. lithotrophicus* ES-1 is the fused monoheme cytochrome porin, Cyc2 (17, 36, 37). *S. lithotrophicus* ES-1 has three isoforms of Cyc2, and all three were detected in both the Fe(II)-citrate and magnetite cultures. One isoform (Slit_0263; Cyc2_1) was one of the most highly expressed proteins in all samples (99.7th percentile; Table 1). The second isoform of the iron oxidase Cyc2 (Slit_0264) was one of the top expressed proteins in the early time-point samples of the fresh and aged synthetic magnetites. Cyc2 expression is high in all the conditions, even ones without measurable amounts of dissolved Fe(II). Together, the results show that while Cyc2 is highly expressed regardless of the presence of dissolved Fe^2+^, the Mto complex is only detected in the presence of solid Fe(II) substrates.

Other proteins that have been previously hypothesized to have a role in iron oxidation were also expressed. *S. lithotrophicus* ES-1 has two gene clusters encoding predicted porin-cytochrome complexes with MHCs, with 18–28 CX_(2-4)_CH motifs, making them much larger than MtoA (34). The proteins encoded by the porin-cytochrome gene cluster Slit_0867–0870 were largely not detected. The proteins encoded by the second porin-cytochrome gene cluster Slit_1446–1449 were detected (with the exception of the predicted extracellular cytochrome, Slit_1448) and had similar levels of expression in all the samples (Table 1). Expression of the proteins encoded by the iron-responsive gene cluster (Slit_1321–1324) identified in Zhou et al. (17) was detected in all samples comparably, where Slit_1323 and Slit_1324 (predicted to be a monoheme and diheme cytochrome *c*, respectively) were expressed >87th percentile (Table 1). The highly expressed and upregulated iron-responsive periplasmic cytochromes identified in Zhou et al. (17) (Slit_1353, Slit_2042, Slit_2657) were also highly abundant in all conditions here (>95th percentile; Table 1).

To identify additional proteins that could be specifically involved in solid Fe(II) oxidation, we compared the time-point with dominantly solid Fe(II) oxidation (late commercial magnetite) to one with solely dissolved Fe^2+^ oxidation (late Fe(II)-citrate). A cluster (Slit_1812–1818) of three cupredoxin domain-containing proteins as well as a SCO1/SenC protein (Slit_1813), a predicted cytochrome (Slit_1812), and a few small hypothetical proteins (Slit_1814–1815) was more highly expressed in the late magnetite cultures compared to the Fe(II)-citrate cultures (Fig. 6; Table S1). Two of the cupredoxin-domain proteins have canonical multicopper oxidase motifs (55) (Slit_1817 and Slit_1818); the third does not (Slit_1816). The protein encoded by Slit_1816 was the most differentially expressed, with significantly higher expression in the late magnetite cultures compared to Fe(II)-citrate. We also identified a periplasmic cytochrome (Slit_2780) with higher expression in the late magnetite cultures than Fe(II)-citrate (Fig. 6; Table S1). These results suggest a possible role for these proteins in the oxidation of solid iron sources.

In all comparisons, there were more proteins with higher expression on the magnetites than on the Fe(II)-citrate samples. However, there were a number of proteins more highly expressed in the Fe(II)-citrate samples. Alternative complex III (Slit_0640–0646) is frequently implicated in iron oxidation pathways and was more highly expressed in the late Fe(II)-citrate cultures compared to the late commercial magnetite culture (Table S2). A cluster of proteins (Slit_0302–0307) was also more highly expressed in the Fe(II)-citrate compared to the late magnetite cultures (Table S2); these proteins may have a function in oxidative stress tolerance. Carbonic anhydrase (Slit_2956) and biotin synthase (BioB) were also more highly expressed in the Fe(II)-citrate cultures (Table S2), suggesting possible differences in CO_2_ metabolism. However, neither form I nor form II RuBisCo were more highly expressed on Fe(II)-citrate, and similar to the results found previously (17), form II was more highly expressed than form I in all cultures (Table S2).

## DISCUSSION

Most of Earth’s iron is mineral-bound, potentially providing a vast source of energy if microbes can obtain electrons from minerals. Chemolithotrophic FeOB could hypothetically grow by mineral oxidation, but to date, there has been scant proof of this ability. Here, we show that a well-studied iron oxidizer *S. lithotrophicus* ES-1 can grow by oxidizing magnetite and constrain the likely enzymatic pathway via proteomics.

Unraveling iron oxidation mechanisms has been hampered by problems with culturing as well as RNA and protein extractions, but recent advances in on-filter, in-cell digestion proteomics methods now enable the study of proteins in low-yield, difficult-to-grow organisms like *S. lithotrophicus* ES-1 (46–48). Previous studies on smectite-grown *S. lithotrophicus* ES-1 included RT-qPCR and proteomics, but proteomic experiments were plagued with a number of issues including interference from iron and filter extractables, as well as the requirement for large volumes of culture to obtain enough cells (7). Proteomics (not transcriptomics) was required to unravel the mechanisms of magnetite oxidation by providing information on the presence of functional protein. Compared to previous studies, this study improved the overall protein detection rate (>78%) with fewer cells (~3–4×10^8^ cells) and improved detection of MHC proteins, which enables us to better evaluate the mechanisms of iron oxidation. We are optimistic that the proteomic pipeline used here will enable the study of other organisms that are difficult to grow.

While it is well-known that FeOB like *S. lithotrophicus* ES-1 can grow by oxidizing dissolved Fe(II), here, we established that it could grow on the solid mixed-valence iron mineral magnetite (Fe^II^Fe^III^_2_O_4_). We carefully followed the progression of iron redox in both dissolved and mineral phases and noted that magnetite oxidation occurred even in the presence of dissolved Fe(II), but the magnetite was never fully oxidized (lowest Fe(II):Fe(III) = 0.3; Fig. 4). Incomplete oxidation of magnetite was similarly observed in experiments with *R. palustris* TIE-1 and enrichment culture KS (1, 3), suggesting that some amount of Fe(II) is not available for either biological or chemical oxidation. Partial oxidation may occur due to kinetic limitations in electron or iron atom diffusion through the magnetite structure (56), resulting in preferential oxidation of the surface as seen for the commercial magnetite (Fig. 4D). Another reason may be that the redox potential of magnetite increases with oxidation (56), causing some of the Fe(II) to be inaccessible due to thermodynamics. Given the various challenges with accessing minerals, it was a surprise that *S. lithotrophicus* ES-1 uses magnetite even in the presence of some dissolved Fe(II). Our results demonstrate that FeOB not only oxidize magnetite and dissolved Fe(II) individually; they can use both solutes and solids simultaneously, opening the question of how common such flexibility may be among other iron oxidizers.

The ability to access electrons from both dissolved and solid Fe(II) may require separate mechanisms appropriate for each type of Fe(II). Oxidation of solid electron donors likely involves the MHC-porin complex MtoAB, which could transfer electrons across the outer membrane. The MtoAB pathway seems to be specifically expressed by *S. lithotrophicus* ES-1 for solid Fe(II) oxidation. In previous studies, when *S. lithotrophicus* ES-1 was grown on Fe(II)-citrate, *mto* transcripts were very low (7, 17). In that study, which also analyzed incomplete proteomes, as well as in our current more comprehensive proteome work, most of the proteins of the Mto pathway were not detected during growth on dissolved Fe(II)-citrate (Table 1; Zhou et al. [7]). However, when grown on solid magnetite, MtoA/MtoB/CymA(ImoA)/Slit_2494 was one of the most significantly enriched sets of proteins (Fig. 6; Table S1), suggesting these proteins are reserved for oxidation of solid Fe(II) sources. This fits with previous research demonstrating that purified MtoA directly interacted with magnetite and was able to extract reactive Fe(II) from within solid ferrite spinel nanoparticles (57). The homologous MtrAB system of iron-reducing *Shewanella* has been shown to be electrochemically reversible and capable of electron uptake (58). Furthermore, the iron-oxidizing homolog in *R. palustris* TIE-1, PioAB, has been shown to play a role in the oxidation of solid electrodes (9, 43), and *R. palustris* can oxidize magnetite (although the proteins involved were not investigated) (1). Combined, these findings strongly imply that the MtoAB MHC-porin complex enables *S. lithotrophicus* to conduct EEU from solid electron donors (magnetite, smectite) and could do so in other organisms as well.

Dissolved Fe(II) is likely oxidized by another iron oxidase of *S. lithotrophicus* ES-1, Cyc2, which is also expressed during growth and oxidation of magnetite. Cyc2 is a monoheme cytochrome fused to a porin, and structural constraints lead to the prediction that Cyc2 must be an oxidase of aqueous Fe^2+^ ions (36). Previous work showed that Cyc2, specifically the first isoform of Cyc2 (Slit_0263), is highly expressed in all growth conditions, including dissolved Fe(II)-citrate, solid Fe(II)-smectite clays, and thiosulfate (7, 17). That continues to be true during growth on magnetite. We hypothesize that dissolved Fe(II) (i.e., Fe^2+^) is the preferred electron donor of *S. lithotrophicus* ES-1, and thus, it maintains readiness to oxidize dissolved Fe(II) regardless of its presence by constitutively expressing Cyc2 at high levels. It is also possible that dissolved Fe(II) at concentrations below our detection limit is being shed from the magnetite and is acting as an electron shuttle, prompting the expression of Cyc2. Another possibility could be that Cyc2 plays a role in iron sensing. In any case, *S. lithotrophicus* ES-1 appears to express its iron oxidases differently. The smaller monoheme cytochrome Cyc2 is expressed under all conditions, while the larger and more energetically expensive complex MtoAB is only expressed when necessary, that is, when a solid electron source is present.

Magnetite oxidation may also involve copper-containing proteins, cupredoxins. Three uncharacterized copper-containing proteins were significantly more highly expressed in the magnetite cultures than the Fe(II)-citrate cultures (Fig. 6; Table S1). Two of these proteins possess typical multicopper oxidase motifs (Slit_1817 and Slit_1818). The third (Slit_1816) does not and is significantly larger, with additional domains similar to adhesions and polysaccharide lyases. These proteins are encoded together in the genome, along with a few smaller proteins, a SCO1/SenC protein, and a hypothetical cytochrome. Similar gene clusters are found in other organisms, mostly other members of the Burkholderiales like *Paraburkholderia* and *Ralstonia* but also in *Anaeromyxobacter* and *Steroidobacteraceae* (Fig. S4). While the roles of these cupredoxin proteins are not known, they are predicted to be extracellular proteins, which would enable access to magnetite particles. Other copper-containing proteins have been reported with ferroxidase (59) and Mn(II)-oxidase activity (60, 61) and play a role in iron oxidation in acidophilic iron oxidizers (62, 63); thus, it is plausible that the cupredoxins identified here are playing a role in solid Fe(II) oxidation in *S. lithotrophicus* ES-1.

Given the growing recognition of microbial mineral oxidation, it will be important to increase our understanding of the mechanisms in order to recognize and trace the activities of mineral-oxidizing microbes. The evidence obtained so far suggests that magnetite and Fe(II)-smectite oxidation in *S. lithotrophicus* ES-1 involves the MtoAB complex, a decaheme cytochrome-porin complex homologous to the *Shewanella* Fe-reductase MtrAB. In studies of *Shewanella*, *Geobacter*, and other FeRB, we have learned that MHCs are well-suited for redox interactions with minerals (64, 65), and their useful characteristics translate well into advantages for oxidizing minerals: (i) when housed in an outer membrane porin, MHCs can transfer electrons across the membrane to or from a mineral. An outer membrane-embedded MHC could either have direct contact with a mineral or transfer to/from extracellular MHC that contact minerals. (ii) Unlike single heme cytochromes, MHCs have wide ranges of redox potentials that overlap with mineral redox potentials, which also span wide ranges and can change as minerals are oxidized and reduced. The MHC MtoA exhibits a range of redox potentials (−400 to +100 mV vs SHE [44, 66]; that overlaps with 10- to 20-nm magnetite (−480 to +50 mV vs. SHE) and smectites (e.g., −600 to +0 mV for SWa-1; −400 to +400 mV for SWy-2 [56, 67, 68];). (iii) MHCs can act as capacitors to store electrons, enabling microbes to continue making energy if there is an interruption in electron supply. This is more likely for minerals, which may be periodically exhausted of electron supply, in contrast to dissolved substrates that tend to be in more constant supply. So, overall, although MHCs are resource-intensive—MtoAB is larger than Cyc2 (1,165 vs ~440 amino acids, 10 heme cofactors vs one)—the investment in biosynthetic energy and resources would enable access to electrons stored in redox-active sedimentary minerals.

The utility of MHCs to FeOB is suggested by the number and diversity of MHCs in known FeOB. More than 60% of the iron-oxidizing Gallionellaceae possess a putative decaheme or larger cytochrome, suggesting many of these iron oxidizers may be able to utilize solid electron donors (45). Many of the Gallionellaceae possess multiple MHC gene clusters. For instance, *S. lithotrophicus* ES-1 encodes MtoAB plus at least two other MHC complexes known as PCC3; these other cytochromes could be used by *S. lithotrophicus* ES-1 to oxidize different solid substrates. It remains to be seen if these are deployed under different conditions individually for distinct substrates or work simultaneously. It will be necessary to further constrain the functional relationships between specific MHCs, minerals, and growth conditions to enable gene- and protein-based tracking of microbial mineral oxidation. MHCs are being increasingly recognized in diverse organisms (69–74), opening the possibility of discovering new mineral-oxidizing organisms and broadening our understanding of the functionality of MHCs.

Overall, our work expands our understanding of how magnetite can promote microbial growth, which has implications for biogeochemical cycling in sediments, aquifers, and rock-hosted environments. In these systems, magnetite can serve as an electron donor to microbes but then can be re-reduced by iron/mineral-reducing microbes (75). Once recharged, the magnetite can be discharged again by FeOB and so on, cycling back and forth, making magnetite a biogeochemical redox buffer that also supports growth and associated C, N, and P transformations. As we increasingly recognize the metabolic flexibility and adaptability of iron oxidizers like *S. lithotrophicus* ES-1 to the varied iron sources on Earth, this will help us understand the active role of iron oxidizers in iron mineral biogeochemical cycling throughout the Earth’s environments.

## MATERIALS AND METHODS

### Magnetite synthesis and preparations

Synthetic magnetite (Fe_3_O_4_) was prepared according to the protocol outlined in Byrne et al. (1). Solutions of 1 M FeCl_2_ and 2M FeCl_3_ were prepared in anoxic 0.3 M HCl. The two solutions were combined and added dropwise into anoxic NaOH (25%) with continuous stirring at 870 rpm in an anaerobic chamber. The black precipitate was collected and washed with anoxic water to remove residual chloride ions. The synthetic magnetite was dried in a desiccator chamber within the anaerobic chamber and then ground with a mortar and pestle under anoxic conditions. This preparation was the fresh synthetic magnetite. For the aged synthetic magnetite, fresh synthetic magnetite was resuspended in anoxic water adjusted to ~pH 10.0 with NaOH. This solution was heated to 95°C for 1 week in a sealed serum bottle in a water bath and then autoclaved for 30 min at 121°C. The aged synthetic magnetite was returned to the anaerobic chamber and then dried and ground as described above. Commercial magnetite (Iron(II,III) oxide; Cas. No. 1317–61-9) was purchased from Sigma-Aldrich (Cat. No. 637106). All types of magnetite were sterilized in an autoclave for 30 min at 121°C as a dry powder under anoxic conditions before use.

### Magnetite characterization

The mineral phase of all dry, sterilized products was confirmed by XRD using a Bruker D8 Powder XRD with Cu Kα radiation. To avoid oxidation during data collection, the samples were loaded into quartz capillary tubes (Charlessupper; outside dimension 1.0 mm) and sealed with silicone in the anaerobic chamber. Data were obtained from 10^o^ to 70^o^ 2*θ* with a step size of 0.05 and an acquisition time of 1 s. The data were collected on autorepeat for at least 15 hours to enhance the diffraction signal. The raw spectra were processed with background subtraction and matched against the database in the DIFFRAC.EVA program. Particle size was calculated based on the Scherrer equation using the full width at half maximum of the six highest intensity peaks (76).

Dissolved and solid Fe measurements were collected using a modified 1,10-phenanthroline assay (77, 78). At selected time-points, samples were taken and then centrifuged in the anaerobic chamber at 13,000 × *g* for 5 min. The supernatant was collected to measure the dissolved Fe^2+^. The precipitates were fully dissolved in 6 M HCl or partially dissolved in 1M HCl under anoxic conditions for 24 hours to determine the ferrous to ferric ratio [Fe(II)/Fe(III)] of the bulk mineral or of the mineral surface (79, 80), respectively. The solutions were diluted 1:4 (1 M HCl) or 1:40 (6 M HCl) with anoxic water. For Fe(II) measurements, 20µL samples were mixed with 80 µL anoxic water, 50µL 0.1% 1,10-phenanthroline, and 50µL 3-M sodium acetate, pH 5.5 in the anaerobic chamber. After a 15min incubation, absorbance was measured at 512 nm and compared to a standard curve. For total Fe measurements, 80 µL of 10% hydroxylamine hydrochloride was used instead of water, and the sample was incubated for 1 hour before addition of the phenanthroline reagent and acetate buffer. Significant differences were determined using a two-tailed, paired *t*-test with a cutoff threshold of 0.05.

### Cultures

*S. lithotrophicus* ES-1 was pre-grown in modified Wolfe’s minimal medium (MWMM) plus trace minerals and vitamins (17, 81, 82), buffered with 20 mM MES pH 6.0 with 10 mM thiosulfate as the electron donor and 2% oxygen as the electron acceptor. Thiosulfate was chosen for the pre-cultures to avoid introducing Fe(III) into experimental reactors. During experiments, synthetic or commercial magnetite (1 g/L; 12.9 mM Fe) with 2% oxygen was utilized as the electron donor and electron acceptor, respectively. The headspace was flushed daily with 2% oxygen/20% carbon dioxide/78% nitrogen. *S. lithotrophicus* ES-1 was also grown in MWMM with 20 mM MES pH 6.0, 5 mM citrate, 2% oxygen headspace, and daily additions of 200 µM FeCl_2_. The cell number was determined by counting SYTO 13-stained cells under fluorescent microscopy using a Petroff–Hausser counting chamber.

### Proteomics

For each sample type, ~3–4 × 10^8^ cells (10–100 mL) were processed following the OFIC digestion protocol described previously (46). In brief, culture was loaded 3 mL at a time onto the cartridges and then centrifuged at 500 x *g* for 1 min. After collecting the total number of cells on the cartridges, cells were incubated with pure methanol at 4°C for 30 min. Afterward, the cartridges were spun to discard the methanol, and the proteins were reduced and alkylated, digested with trypsin (Promega, Madison, WI), eluted, and then desalted using C18-based StageTips (CDS Analytical, Oxford, PA) as described previously (46). The LC-MS/MS analysis was performed using an Ultimate 3000 RSLCnano system and Orbitrap Eclipse mass spectrometer installed with FAIMS Pro Interface (ThermoScientific) also as described previously (46).

For proteome quantitation, raw MS data were processed using MaxQuant (83) and Andromeda software suite (version 2.4.2.0). The protein database of *S. lithotrophicus* ES-1 (taxonomy_id:580332; 2,978 protein sequences) was downloaded from the UniProtKB website (https://www.uniprot.org/). The enzyme specificity was set to 'Trypsin'; variable modifications include oxidation of methionine and acetyl (protein N-terminus); fixed modification includes carbamidomethylation of cysteine. The maximum missed cleavage sites were set to 2, and the minimum number of amino acids required for peptide identification was 7. The false discovery rate (FDR) was set to 1% for protein and peptide identifications. MaxLFQ function embedded in MaxQuant was enabled for label-free quantitation, and the LFQ minimum ratio count was set to 1. Proteins identified as reverse hits, potential contaminants, or only by site-modification were filtered out from the “proteinGroups.txt” output file. The LFQ values were log_2_ transformed, filtered by at least two valid values out of three replicates in at least one group, and imputed using the default “normal distribution” method in Perseus (version 2.0.6.0) (84).

### Analysis tools

Venn diagrams were created using goodcalculators.com/venn-diagram-maker. Heatmaps were made in R using ggplot2 (85). Gene cluster comparisons were performed using cblaster and clinker https://cagecat.bioinformatics.nl/ (86, 87). Protein subcellular localization predicted using (PSORTb v3.0.3 https://www.psort.org/psortb/ (88). All the statistical analyses, including the Student’s *t*-test with permutation-based FDR, were performed using Perseus (version 2.0.6.0) (84).

## Data Availability

The MS raw files associated with this study have been deposited to the MassIVE server (https://massive.ucsd.edu/) with the data set identifier MSV000093770 and are publicly available. Unprocessed protein intensities and iBAQ values (Table S3) and processed pairwise comparisons (Table S4) are available as part of the supplemental material.

## SUPPLEMENTAL MATERIAL

Supplemental material aem.01865-24-s0001.pdf Figures S1 to S4 and Tables S1 and S2.

Table S3 aem.01865-24-s0002.xlsx Unprocessed LQF intensities and iBAQ values.

Table S4 aem.01865-24-s0003.xlsx Processed pairwise comparisons.

ASM does not own the copyrights to Supplemental Material that may be linked to, or accessed through, an article. The authors have granted ASM a non-exclusive, world-wide license to publish the Supplemental Material files. Please contact the corresponding author directly for reuse.
