# Supplementary material for: Microbial magnetite oxidation via MtoAB porin-multiheme cytochrome complex in Sideroxydans lithotrophicus ES-1
Source: Appl Environ Microbiol. 2025 Mar 5;91(4):e01865-24. doi: 10.1128/aem.01865-24 (PMC12016527; doi:10.1128/aem.01865-24)
Supplement: Supplemental material — Figures S1 to S4 and Tables S1 and S2. [file aem.01865-24-s0001.pdf]

Microbial magnetite oxidation via MtoAB porin-multiheme cytochrome complex in *Sideroxydans lithotrophicus* ES-1

Jessica L. Keffer[1*], Nanqing Zhou[2+], Danielle D. Rushworth[1], Yanbao Yu[3], Clara S. Chan[1,2,*]

[1]Department of Earth Sciences, University of Delaware, Newark, DE

[2]School of Marine Science and Policy, University of Delaware, Newark, DE

[3]Department of Chemistry and Biochemistry, University of Delaware, Newark, DE

[+]current address: Department of Civil and Environmental Engineering, Northwestern University, Evanston, IL

[*]corresponding authors (jlkeffer@udel.edu and cschan@udel.edu)

List of Figures and Tables:

Figure S1 – page 2

Figure S2 – page 3

Figure S3 – page 4

Figure S4 – page 5

Table S1 – page 6

Table S2 – page 7

Table S3 – Unprocessed LQF intensities and iBAQ values (xlsx file)

Table S4 – Processed pairwise comparisons (xlsx file)

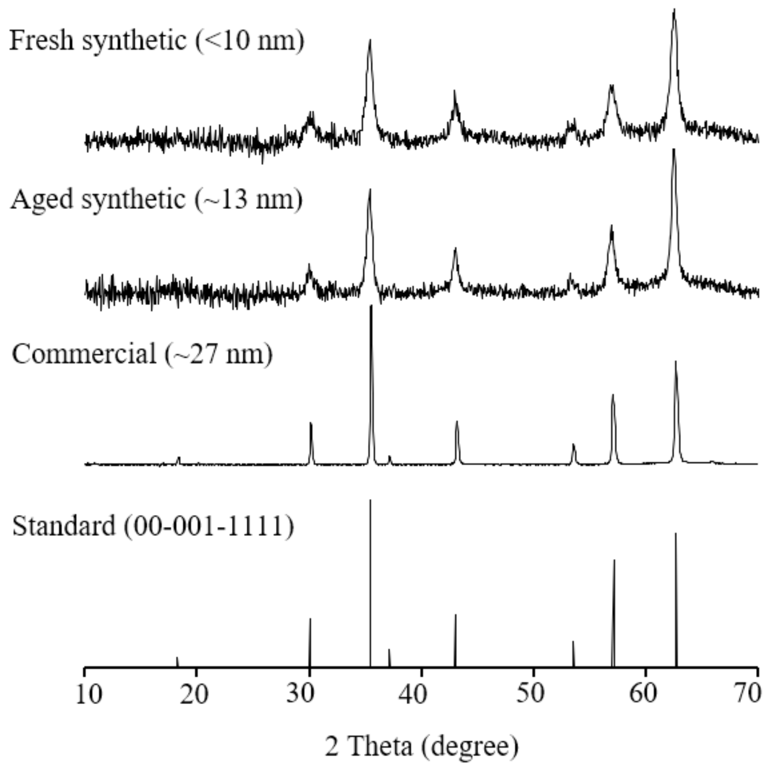

Figure S1. XRD patterns of (from top to bottom): fresh synthetic magnetite, aged synthetic magnetite, commercial magnetite, and magnetite (Fe_3O_4) standard 00-001-1111. Calculated particles sizes in parentheses.

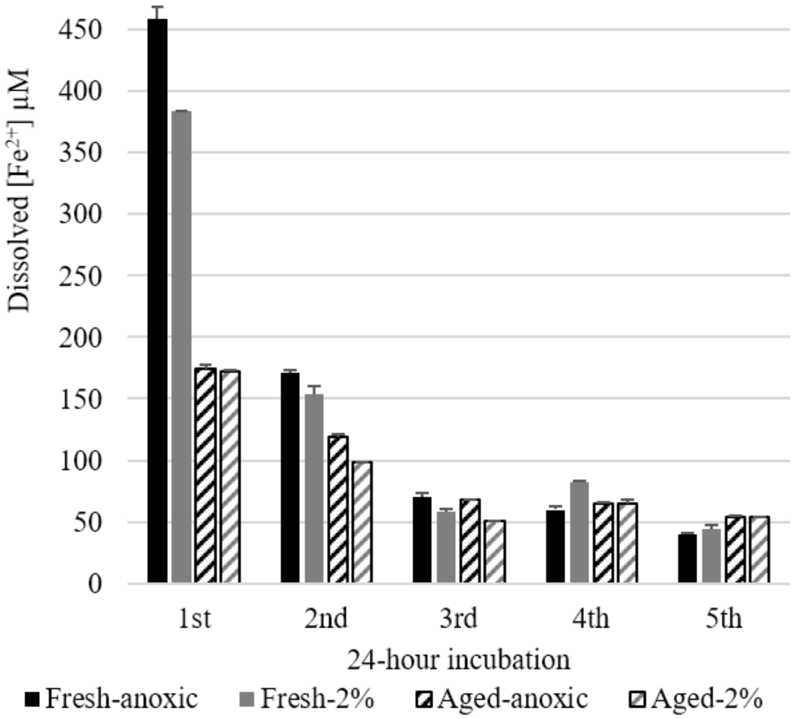

Figure S2. Dissolved Fe^{2+} released under anoxic (black) or 2% oxygen (gray) conditions in 20 mM MES pH 6.0 from different magnetite types: fresh synthetic magnetite (solid bars), aged synthetic magnetite (hashed bars). Commercial magnetite was not measured since its first release is below the limit of detection (<10 μM). Error bars are + one standard deviation for replicates.

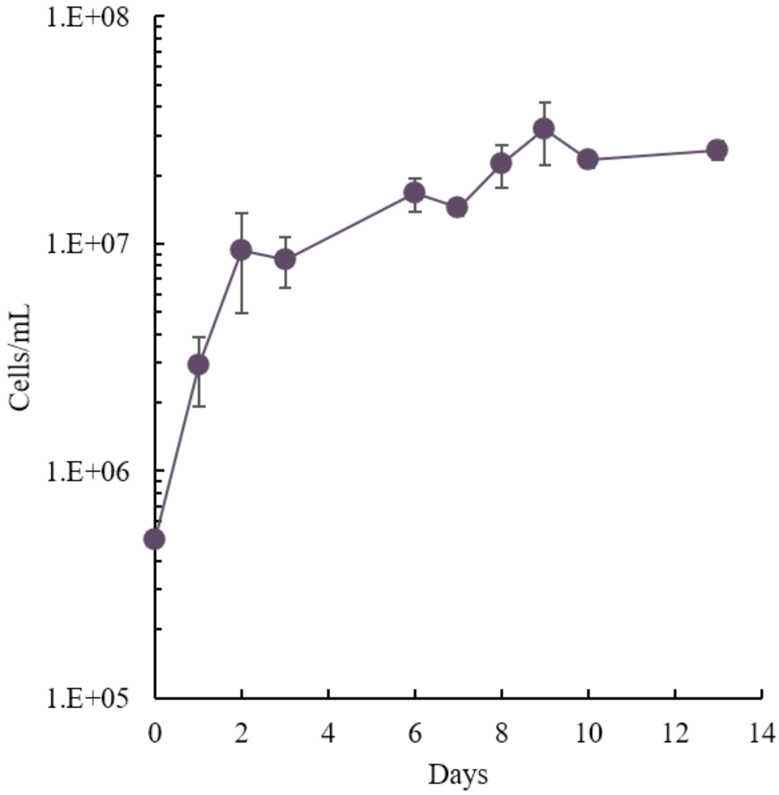

Figure S3. *S. lithotrophicus* ES-1 growth on Fe(II)-citrate (200 µM/day). Error bars are ± one standard deviation for replicates.

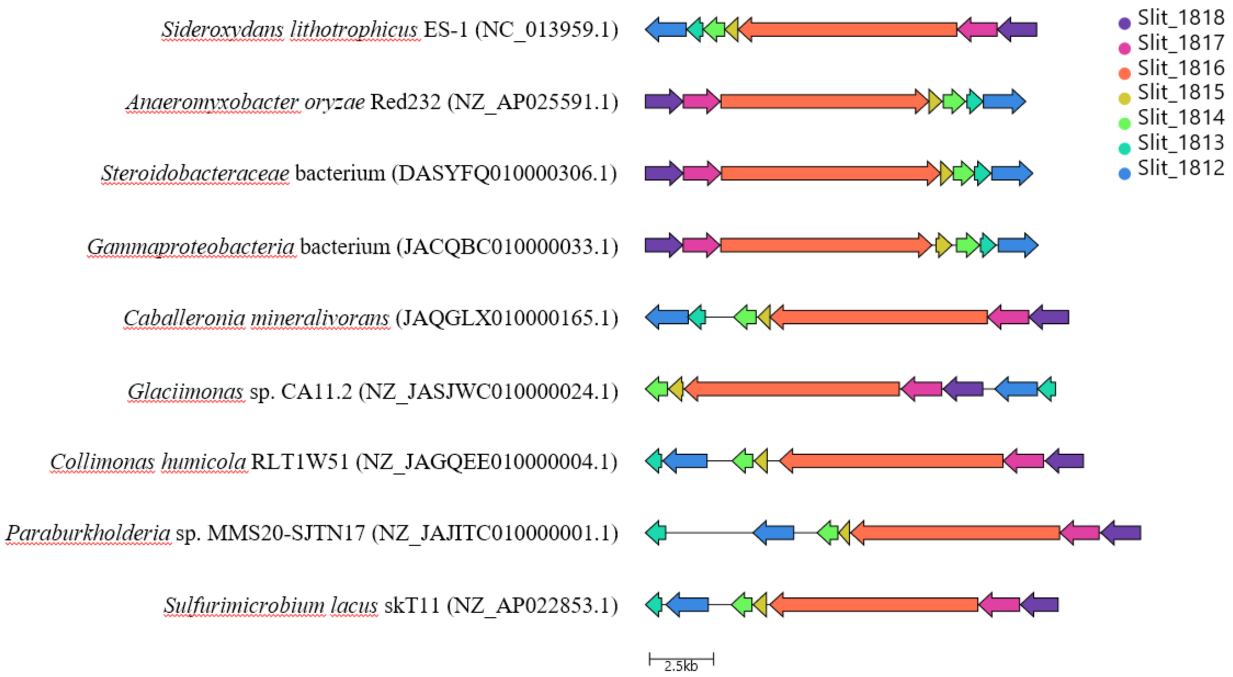

Figure S4. Gene cluster comparison between *Sideroxydans lithotrophicus* ES-1 and other selected organisms possessing a similar three cupredoxin-containing cluster. Accession number in parentheses. Figure generated using cblaster and clinker (https://cagecat.bioinformatics.nl).

Table S1. p-values for each log_2 fold change presented in Figure 6.

Protein	Fresh Mag	Aged Mag	Comm Mag
Cyc2_1	0.0006	0.00006	0.03
Cyc2_2	0.0007	0.00006	0.5
Cyc2_3	0.008	0.0001	0.03
Cyt-c (Slit_2494)	0.000007	0.000002	0.0005
CymA/ImoA	0.00002	0.00005	0.02
MtoB	0.0007	0.001	0.005
MtoA	0.0005	0.001	0.0008
Slit_1812	0.00004	0.0005	
Slit_1814	0.00003	0.000003	0.02
Slit_1815	0.0002	0.0000006	0.3
Slit_1816	0.00008	0.00003	0.001
Slit_1817	0.0003	0.0003	0.03
Slit_1818	0.004	0.001	
Slit_2780	0.001	0.0008	0.02

Mag – magnetite; Comm – commerical; Cyt - cytochrome

Table S2. Maximum percentile of protein expression based on iBAQ values and log_2 fold change for late Fe(II)-citrate cultures compared to commercial magnetite.

Protein Name	Locus Tag	Fe-cit (Late)	Fresh Mag (Late)	Aged Mag (Late)	Comm Mag (Late)	Log2 Foldchange (FC/CM)	p-value
ACIII	Slit_0640	83.5	79.9	83.4	21	2.53876	0.004
ACIII	Slit_0641	95.1	93.8	95.1	74.3	2.20014	0.02
ACIII	Slit_0642	94.8	91.3	94.5	72.7	2.30073	0.03
ACIII	Slit_0643	74.8	62	52.2	27.5	3.22756	0.004
ACIII	Slit_0644	96.4	95.1	97.4	76.4	2.21245	0.02
ACIII	Slit_0645	78.4	73.1	74.7	36.8	2.16156	0.04
Rubrerythrin	Slit_0302	99.9	92.9	93.3	94.4	3.65931	0.005
Uncharacterized	Slit_0303	86.4	38.9	38.7	41.2	2.70022	0.006
Uncharacterized	Slit_0304	93.2	13.1	41.6	19.1	6.11879	0.003
Ferritin family	Slit_0305	99.4	87.5	91.7	88.2	2.98962	0.01
NnrS family	Slit_0307	45	0	6.7	0	1.31085	0.05
Carbonic anhydrase	Slit_2956	84.7	0	0	0	4.99871	0.0007
Biotin synthase	bioB	71.6	13.6	13.2	13.3	3.31605	0.007
cbbM	Slit_0022	97.6	96.7	98	97.5	-0.56375	0.4
cbbL	cbbL	10.1	7.6	0	12.2	-1.18568	0.4
cbbS	Slit_0986	10.3	0	0	30.8	-2.00883	0.04

Fe-cit – Fe(II)-citrate; Mag – magnetite; Comm – commercial; FC – late Fe(II)-citrate; CM – late commerical magnetite
